# Comparative Genomics Reveals a Significant Sequence Variability of Myticin Genes in *Mytilus galloprovincialis*

**DOI:** 10.3390/biom10060943

**Published:** 2020-06-22

**Authors:** Magalí Rey-Campos, Beatriz Novoa, Alberto Pallavicini, Marco Gerdol, Antonio Figueras

**Affiliations:** 1 Institute of Marine Research (IIM), CSIC, Eduardo Cabello 6, 36208 Vigo, Spain; mrey@iim.csic.es (M.R.-C.); beatriznovoa@iim.csic.es (B.N.); 2 Department of Life Sciences, University of Trieste, Via Giorgieri 5, 34127 Trieste, Italy; pallavic@units.it; 3 National Institute of Oceanography and Applied Geophysics—OGS, via Auguste Piccard, 54, 34151 Trieste, Italy

**Keywords:** *Mytilus galloprovincialis*, myticins, mussel genome, RNA-seq, positive and negative selection, promoter, antimicrobial peptide

## Abstract

Myticins are cysteine-rich antimicrobial peptides highly expressed in hemocytes of *Mytilus galloprovincialis*. Along with other antimicrobial peptides (AMPs), myticins are potent effectors in the mussel immune response to pathogenic infections. As intertidal filter-feeders, mussels are constantly exposed to mutable environmental conditions, as well as to the presence of many pathogens, and myticins may be key players in the great ability of these organisms to withstand these conditions. These AMPs are known to be characterized by a remarkable sequence diversity, which was further explored in this work, thanks to the analysis of the recently released genome sequencing data from 16 specimens. Altogether, we collected 120 different sequence variants, evidencing the important impact of presence/absence variation and positive selection in shaping the repertoire of myticin genes of each individual. From a functional point of view, both the isoelectric point (pI) and the predicted charge of the mature peptide show unusually low values compared with other cysteine-rich AMPs, reinforcing previous observations that myticins may have accessory functions not directly linked with microbe killing. Finally, we report the presence of highly conserved regulatory elements in the promoter region of myticin genes, which might explain their strong hemocyte-specific expression.

## 1. Introduction

Myticins are cysteine-rich antimicrobial peptides, discovered in hemocytes and plasma of *Mytilus galloprovincialis* in 1999 [1]. Since then, information about their gene and protein structure, sequence diversity, and function have been reported, mainly for the highly variable myticin C [2,3,4,5,6]. However, these studies have probably raised so far more questions about the function and evolution of myticins than they solved.

*M. galloprovincialis* is a filter-feeding invertebrate, incessantly exposed to pathogens and pollutants [7]. Despite this, these animals show an incredible capability to resist adverse conditions, as evidenced by the lack of massive mortalities reported in the natural environment [8,9], in contrast to other bivalves such as clams [10] or oysters [11]. This fact can be explained by the effectiveness of mussel immunity, which primarily relies on hemocytes, which produce a significant variety of antimicrobial peptides (AMPs). Transcriptomic data revealed that hemocytes constitutively express very high levels of myticins [2,12] and other small cationic and cysteine-rich peptides such as defensins [13], mytilins [14], and mytimycins [15]. Altogether, these molecules constitute a complex repertoire of AMPs that is thought to have been developed by evolution as an effective defense system against pathogens present in the marine environment.

Like other mussel AMPs, myticin genes comprise 4 exons and 3 introns. The first exon is the smallest one and it only includes the non-coding 5’UTR region. The second, third, and fourth exons encode the signal peptide (about 20 amino acids), the mature peptide (about 40 amino acids), and the C-terminal region (about 40 amino acids), respectively [4]. Like several other AMPs, myticins are therefore produced as inactive precursors, stored in hemocyte granules and activated by the proteolytic cleavage of the C-terminal region upon infection [6]. The very first studies carried out on these molecules allowed for the identification of different myticin variants, enabling a first categorization of these sequences among three main isoforms (A, B, and C) [1,2]. The three isoforms showed slightly different primary sequences and similar lengths, with the exception of a 4-amino-acid-long insertion in the C-terminal part of myticin C. However, further studies have added additional complexity to this picture with the use of massive sequencing [16] and the aforementioned classification now appears to be outdated.

Despite the variability of mussel myticins, some amino acids found in their mature peptide are highly conserved. In particular, eight highly conserved cysteine residues are engaged in four intramolecular disulfide bridges, which define the typical cysteine-stabilized alpha-beta (CSαβ) motif shared by myticins and other AMPs, such as defensins and mytilins [17].

From a functional point of view, myticin A and B have been defined as antibacterial and antifungal peptides [1]. On the other hand, myticin C, the isoform subjected so far to more intense studies, has been linked not just to antibacterial [18], but also to antiviral and cytokine-like functions [5,6]. The antiviral activity of myticin C was evidenced against fish viruses (VHSV and IPNV) [5], oyster herpesvirus (OsHV-1), and even human herpesvirus (HSV-1 and HSV-2) [6]. Moreover, the chemotactic activity defined in 2011 by Balseiro et al. [5] attributed myticin C a new chemokine role, which is further supported by our recent finding that these peptides are involved in tissue injury and regeneration processes in *M. galloprovincialis* [19,20].

The main objective of this work was to shed some light on whether the remarkable intraspecific sequence variability of myticins derives from the complex genomic architecture of *M. galloprovincialis* or from RNA editing, exploiting the new information obtained from the mussel genome and the massive resequencing data of 16 additional individuals [21]. The high variability of these AMPs and other immune effectors may represent a key factor in explaining the great evolutionary success of this species.

## 2. Materials and Methods

### 2.1. Searching, Screening, and Identifying M. galloprovincialis Myticins

The recently published reference mussel genome [21], and the resequenced genome assemblies of 16 additional different individual genomes from Galicia and Italy (9 males and 7 females) were screened for the presence of myticins gene variants. In this work, these genomic resources will be labeled as follows: LOLA (the reference genome), PURA, GALF1, GALF2, GALF3, GALM1, GALM2, GALM3, GALM6, GALM11 (from Galicia), ITAF1, ITAF2, ITAF3, ITAM1, ITAM2, and ITAM3 (from Italy). “F” and “M” indicate female and male mussels, respectively; note that LOLA and PURA are female individuals. Briefly, as detailed in the original paper [21], the mussel reference genome was assembled through a hybrid multi-step process, which included 2 × 101 bp paired-end, mate-pair, and fosmid-end Illumina reads, generated on a HiSeq2000 platform, as well as long PacBio reads, generated on a Sequel platform. Overall, the Illumina PE and PacBio sequencing outputs accounted for ~110 X and ~10 X coverage, respectively. The final assembly underwent multiple rounds of scaffolding, decontamination from exogenous contaminants, and removal of duplicated haplotype blocks to obtain a haploid reference assembly. The de novo assemblies of the resequenced individuals were obtained with the CLC Genomics Workbench 20.0.3 (Qiagen, Hilden, Germany) starting from 2 × 150 bp Illumina reads, generated with a HiSeq2500 platform, which accounted for ~30–35 X coverage.

All 16 genome assemblies were used to create BLAST databases. Previously described myticins [1,2], and specifically the third exon (mature peptide coding region) were used as a query to perform tBLASTn searches against each genome assembly, with an e-value threshold of 1 × 10^−5^, using the CLC Genomics Workbench 20.0.3 (Qiagen, Hilden, Germany). The resulting hits were manually checked to verify that they were indeed myticins (myticin database available in File S1). The reliability of the sequences obtained was verified by the visual inspection of read mapping data obtained with strict mapping thresholds (set with the CLC Genomics Workbench 20.0.3) to ensure the lack of sequencing or assembly errors.

The in silico translated exon 3 sequences of all obtained sequences were aligned using MUSCLE in the MEGA-X Software environment [22].

All the nucleotide sequences were clustered by similarity using the CD-HIT server [23], setting a sequence identity cut-off of 0.95. A consensus sequence was then established for each cluster, enabling further downstream analyses.

### 2.2. Phylogenetic Analysis

The whole set of nucleotide sequences was taken into account to find the best suitable molecular model of evolution. jModelTest [24,25] was the software used for this purpose, and the choice of the best-fitting model, i.e., a Jukes–Cantor model [26], was performed based on the corrected Akaike information criterion.

A Bayesian inference analysis was run with a Markov chain Monte Carlo approach using the MrBayes v3.2.7 Software [27]. Two independent analyses with four chains each were run in parallel for 600,000 generations until the effective sample size parameter estimated for all the parameters of the model reached a value >200. The resulting phylogenetic tree was graphically represented using FigTree v1.4.4. [28]. By the same approach, a phylogenetic analysis was also performed using the established consensus sequences for each of the clusters of mussel myticins.

### 2.3. Isoelectrical Point

The N-terminal end of the mature peptides was predicted based on the detection of the signal peptide cleavage site with SignalP v3.0 [29]. Due to the unknown nature of the protease involved in the cleavage of the C-terminal region of myticin precursors, the putative C-terminal end of the mature peptide was identified based on the alignment with the known mature peptides previously described by other authors [1].

The isoelectric point (pI) and the charge of the mature peptide (at cytoplasmic pH = 7.4) of all the conventional myticins (myticins that display the usual 8-cysteine array in the mature peptide) was calculated using the Isoelectric Point Calculator Software [30]. Moreover, the pI distribution of the complete peptide of the three classically defined myticins A, B, and C was analyzed through the calculation of the average pI based on a sliding window of 15 amino acids.

### 2.4. Positive and Negative Selection Analysis

The codon-aligned nucleotide sequences of the exon 3 of the whole set of conventional myticins were analyzed to detect sites evolving under episodic positive selection with the MEME algorithm [31], as well as pervasive positive/negative selection using FEL [32], FUBAR [33], and SLAC [32] algorithms. A similar analysis was also performed on a subset of sequences belonging to the myticin C clade only. These analyses were performed using Datamonkey Adaptive Evolution Server [34]. The predicted three-dimensional structure of mature peptide of myticin C was obtained from a previous publication [35] and modified with Chimera 1.14 [36] to highlight sites under significant positive and negative selection (i.e., p-value lower than the 0.1 threshold).

### 2.5. Promoter Analysis

The previously identified exon 3 matches were used as a seed to extend the reconstruction of full myticin genes from the genome assemblies, retrieving the sequences of exons 1, 2, and 4, whenever possible. The Genie tool [37] was used to predict the 5’ splicing acceptor sites and the 3’ splicing donor sites, and thereby to define the boundaries of cited exons, with the aid of the alignment between the genomic DNA and cDNA sequences obtained by previous studies, whenever available. Once the full gene structure was appropriately annotated (15 out of 32 clusters, full sequences in File S2), we extracted a fragment of 500 bp upstream of the first exon to perform a promoter analysis by searching for conserved ungapped motifs shared by most myticin genes. This length threshold was selected as a compromise between the inclusion of a significant number of sequences in the analysis and the possibility to explore a biologically meaningful sequence context (i.e., the core and proximal promoter), based on the suggestions provided by Zia and Moses [38] to limit false positive detection. Obviously, the downside of this approach, also linked with the relatively high fragmentation rate of the mussel genome reassemblies, was the impossibility of investigating the presence of distal regulating elements present upstream or downstream of the transcription start site. The de novo motif-finding analyses were run with MEME Suite 5.1.1 [39], selecting the classic motif discovery mode and setting the accepted length of such motifs between 6 and 30 bp.

The significant motifs obtained (combined match p-value lower than 1e-15) were kept for a further search in the LOLA assembly (the reference genome assembly, [21]) in order to determine if the motifs identified in the myticin promoter region were also associated with other mussel genes. For this, the motif search tool of CLC Genomics Workbench 20.0.3 was used. A list containing the 10 consensus motif sequences of myticin was created and the subsequent search was performed with 70% accuracy.

### 2.6. Genomic vs. Transcriptomic Data

In addition to the genome of LOLA, its transcriptome was also available [21]. LOLA myticin sequences found in the genome (detailed location in the File S3) were compared to all sequences present in the transcriptome (the approach to search the sequences in the transcriptome assembly was the same as the genomic approach, previously described). Genome reads were mapped to the myticin sequences found in the transcriptome in order to determine differences between the DNA and RNA of the same individual. The mapping parameters were set to be highly restrictive in order to only allow perfect matches (length fraction = 1 and similarity fraction = 1). The mapping files obtained were then visually inspected to detect regions with no read coverage, which could indicate mismatches between the genomic DNA and mRNA sequences and pinpoint the presence of sites subjected to RNA editing.

### 2.7. Expression Analysis

Taking advantage of the transcriptomic information available in the Sequence Read Archive, National Center for Biotechnology Information (SRA-NCBI), 6 different transcriptome assemblies of *M. galloprovincialis* (from different geographic locations and different tissues) were used to find evidence of expression of all the different clusters of myticin. These transcriptomes are the following: PRJNA88481 (digestive gland, which also included unpublished gill data available at the University of Trieste), PRJNA525609 (mantle), PRJNA249058 (whole body), PRJNA484309 (gill and mantle), PRJNA230138 (hemocytes, mantle, muscle, and gill) and PRJNA466718 (hemocytes). These resources were screened with the tBLASTn approach, using the mature peptide regions of the aforementioned myticin clusters as a query, as previously described. In this case, the finding of a match sharing >95% identity with the query sequence was considered as an evidence of the expression of a myticin variant belonging to the underlying sequence cluster. All the additional contigs identified that displayed >5% divergence compared with the established clusters were considered as belonging to new unreported clusters and added to the myticin sequence dataset (File S1).

## 3. Results

### 3.1. Searching, Screening, and Identifying M. galloprovincialis Myticins

**Figure 1:**
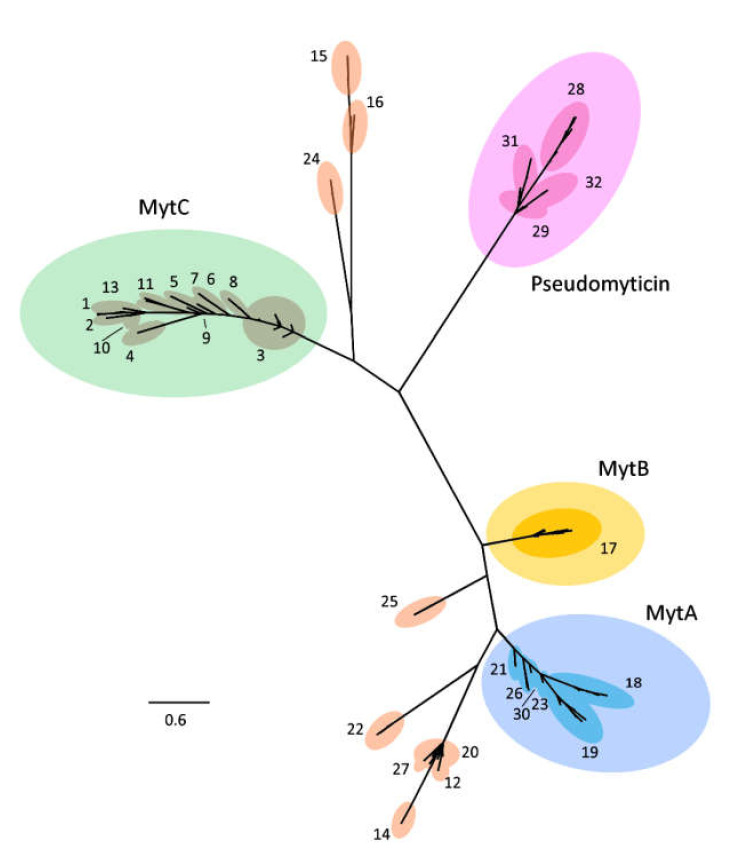
**Phylogenetic analysis.** Exon 3 of 120 myticin sequences obtained from 16 genomes were subjected to multiple sequence alignment and analyzed with Bayesian phylogenetic inference. The employed evolution model was the Jukes and Cantor model (JC). Numbers at termini correspond to the 32 major groups found by CD-HIT (based on a pairwise sequence identity >95%). Green (MytC), yellow (MytB), blue (MytA), and purple (pseudomyticin) ellipses indicate the variants falling within the four main described groups of myticins and pseudomyticins.

A total of 120 different nucleotide sequences encoding myticins were found in the 16 mussel genome assemblies. From these 120 sequences, 93 were conventional myticins (myticins that display the usual 8-cysteine array in the mature peptide), 9 were pseudogenes (sequences that incorporate a STOP codon which interrupts the open reading frame), and 18 were pseudomyticins (sequences that keep the most of the structure of myticins but lose a pair of cysteines) (File S1). All the sequences were clustered based on an identity percentage threshold of 95%, obtaining a total of 32 different clusters (File S1). The Bayesian tree of all the 120 myticin variants (alignment available in the File S4) identified in this study (Figure 1) displayed a remarkable sequence diversification, with a subdivision of classical myticins (A, B, and C) and pseudomyticins, as well as other newly reported myticins belonging to intermediate branches.

### 3.2. Phylogenetic Analysis

**Figure 2:**
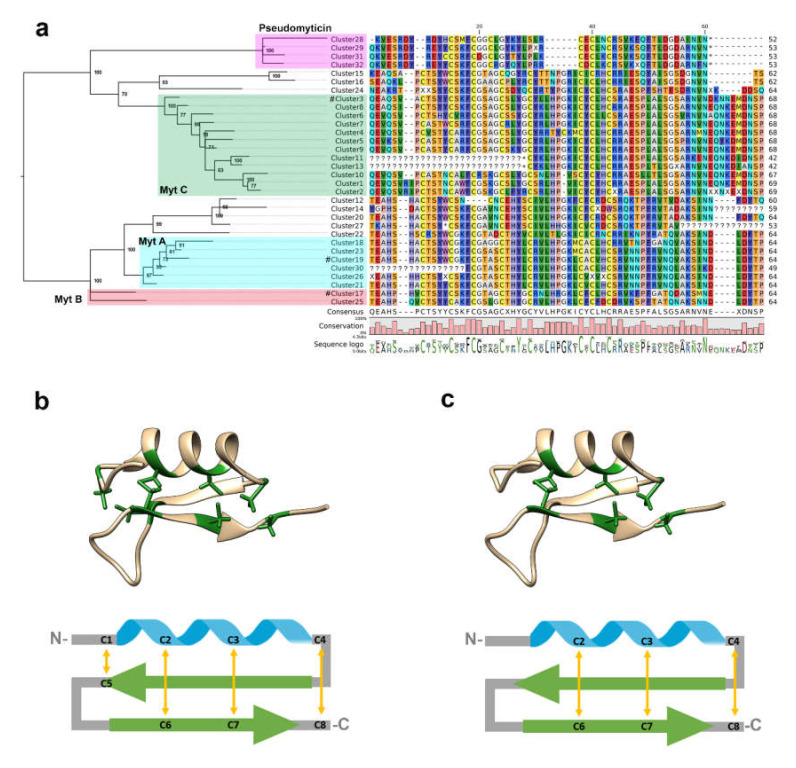
**Clusters phylogenetic analysis and alignment.** (**a**) A consensus sequence of each of the 32 myticin clusters (exon 3) was used to run the phylogenetic analysis and build the alignment. ? represents parts of sequences that likely resulted from an exon truncation event, thereby not finding any significant similarity with the classical myticin structure. X represents an ambiguous consensus sequence for a given codon in a given sequence cluster, resulting in an undetermined amino acid residue. * represents STOP codons. Green (MytC), red (MytB), blue (MytA), and purple (pseudomyticin) squares show the position of the four main described groups of myticins and pseudomyticins. # represents previously published Myt A/B/C sequences. (**b**,**c**) show the predicted secondary structure (green color highlights the cysteine positions) and the putative interaction of cysteines engaged in the formation of the disulfide bonds (yellow arrows) of conventional myticins and pseudomyticins, respectively.

For simplicity’s sake, a simplified version of myticin phylogeny, showing the evolutionary relationship among myticin clusters, is displayed in Figure 2a. The same figure also displays an alignment of the consensus sequence of the 32 myticin clusters, which allows us to note the greatest differences between pseudomyticins and the rest of myticins. This diversified group has lost 2 out of 8 characteristic cysteines of this gene family, namely Cys1 and Cys5, which are expected to be engaged in one of the four disulfide bonds of the CSαβ structural scaffold of myticins. Two additional panels of Figure 2 show the expected disulfide array of conventional myticins (Figure 2b) and pseudomyticins (Figure 2c).

### 3.3. Presence/Absence Variation

An evaluation of the presence/absence of all 120 sequences was performed in the 16 mussel genomes (File S5). On average, each mussel genome showed 11 myticin different sequences, of which around 6 were exclusively found in one out of the 16 individuals analyzed. The presence/absence matrix highlights the great inter-individual diversity in the repertoire of myticins of each individual, which results in a virtually unique collection of variants in each mussel. The sequences located at the top of the matrix are those which displayed the highest frequency of occurrence (2, 98_PM, 1, and 18). Even so, none of them was present in all the genomes analyzed.

**Figure 3:**
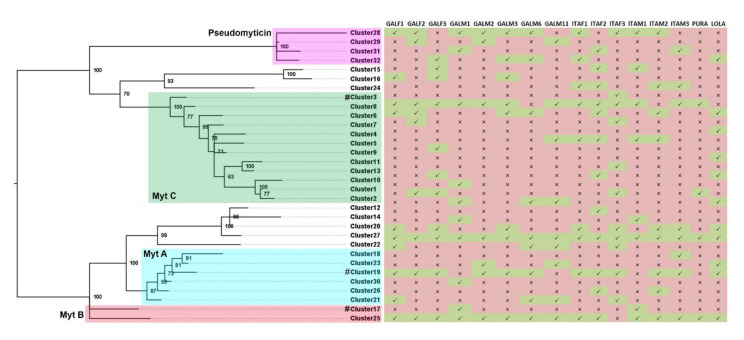
**Presence/absence evaluation of the 32 clusters of myticin.** The matrix shows presence/absence of each cluster in the 16 mussel genomes. Green (MytC), red (MytB), blue (MytA), and purple (pseudomyticin) squares show the position of the four main described groups of myticins and pseudomyticins. # represents previously published Myt A/B/C sequences.

As several of the 120 variants identified only displayed minor differences in pairwise comparisons, we cannot exclude that they represent polymorphic alleles of the same gene. Our clusterization approach allowed us to take into account these uncertainties in the ascertainment of presence/absence variation, identifying several groups of myticins shared by most of the 16 analyzed genomes and others which were just found in a low number of individuals, or were even exclusively present in a single one (Figure 3). It could be observed as at least one representative of each of the four major groups of myticins (A, B, C, and pseudomyticins) was present in all or almost all the genomes (clusters 8, 19, 25, and 28). Of all sequence clusters, only cluster 27 was present in all individuals.

### 3.4. Isoelectric Point

**Figure 4:**
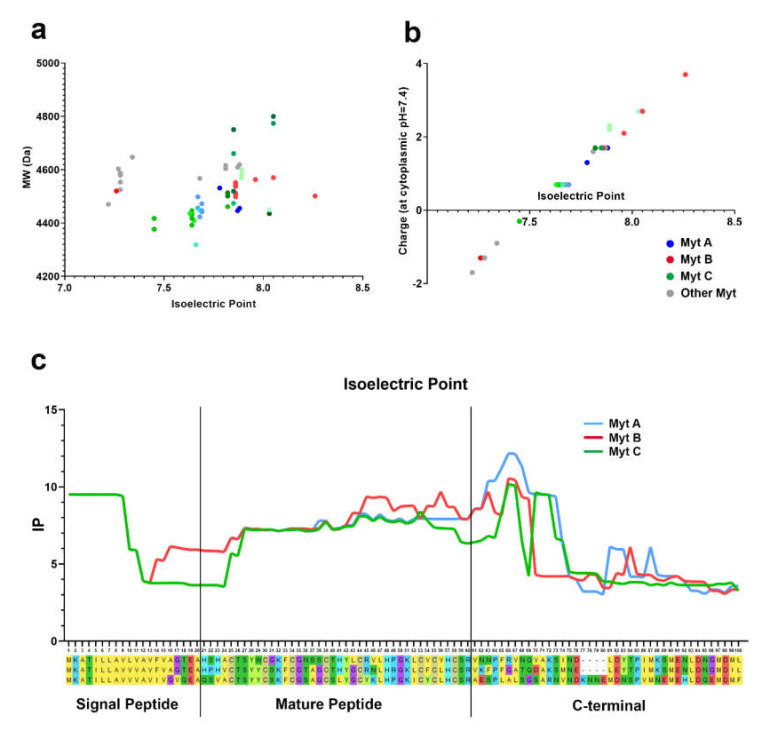
**Isoelectric Point.** (**a**) Isoelectric point (X axis) and molecular weight (Y axis) of the mature peptide of each conventional myticins (71 defined mature peptides, obtained by removing redundant amino acid sequences and pseudogenes). (**b**) Isoelectric point (X axis) and charge at pH = 7.4 (Y axis) of the mature peptide of each conventional myticins (71 defined mature peptide). (**c**) Isoelectric point of the whole sequence of one representative of each conventional myticin (A, B, and C). The isoelectric point distribution was analyzed through the calculation of the average isoelectric point based on a sliding window of 15 amino acids.

The isoelectric point and predicted charge at cytoplasmic pH (i.e., 7.4) of all the conventional myticin sequences obtained (mature peptide) are reported in File S6. Figure 4a shows that all myticins display very narrow variations in terms of pI, which varies between 7 and 8. It can also be observed that neither pI, nor the molecular weight of the mature peptide depend on the myticin isoform. The charge of the mature peptides is usually slightly positive, varying from −2 to 4 (Figure 4b), with no remarkable differences between the different myticin isoforms.

The sliding-window analysis of pI along the whole peptide, carried out on the three representative precursor peptides of myticin A, B, and C (Figure 4c), showed very similar profiles, with a stable value across the mature peptide, and just a slight decrease in the C-terminal part of the sequence, of a much smaller entity than previously observed in other mussel AMPs, such as mytilins [40].

### 3.5. Positive and Negative Selection Analysis

**Figure 5:**
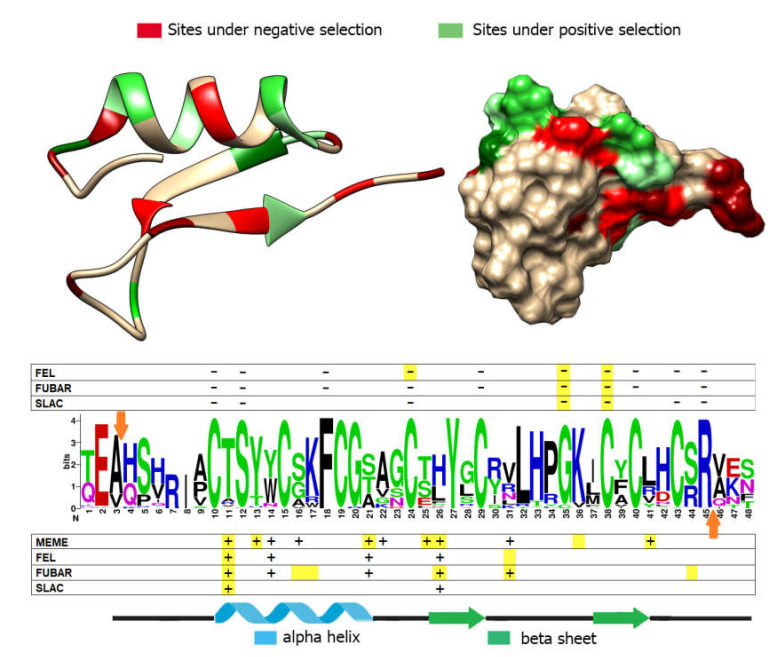
**Positive and negative selection analysis.** Exon 3 of all conventional myticins were selected to perform the analysis, discarding pseudogenic sequences. Four prediction models have been used (MEME, FEL, FUBAR, and SLAC). Positively selected sites (+) and negatively selection sites (−) identified in the complete myticin sequence dataset are both shown on the graph. Positively and negatively selected sites identified in the myticin C sequence clade are marked with a yellow background. Orange arrows point to the beginning and end sites of the mature peptide. The ribbon structure and the molecular surface, highlighting in red the sites under negative selection and in green the sites under positive selection, are also shown [from 35].

The selection analysis identified several sites subject to positive or negative selection in the mature peptide region. Despite some minor differences, the various tests were concordant in recognizing multiple sites under pervasive purifying selection (Figure 5). These sites match with six of the cysteines that form the characteristic disulfide array of myticins. Specifically, Cys-1, -4, -5, -6, -7, and -8 are under a strong negative selection. Another interesting case of negative selection is the arginine that marks the end of the mature peptide, which may be recognized as the signal for proteolytic cleavage of the precursor protein. Three additional negatively selected sites of interest that emerged from this analysis are a conserved glycine found in a tight turn which connects the two antiparallel beta sheets, and a serine and a phenylalanine residue found in the alpha helical region. The tests also identified a total of 5 sites evolving under significant positive selection (4 by FEL, 5 by FUBAR, and 2 by SLAC). MEME further indicated the possibility that 10 out of the 42 amino acids in the myticin mature peptide might have undergone episodic positive selection. The functional and structural role of these hypervariable sites is presently unknown. The same analysis, run on the sequences belonging to the highly variable myticin C clade only, revealed a good overlap of selected sites compared with the full sequence dataset, supporting the reliability of the results described above and pointing out that the signals obtained were not just the result of ancestral divergence among paralogs.

### 3.6. Promoter Analysis

**Figure 6:**
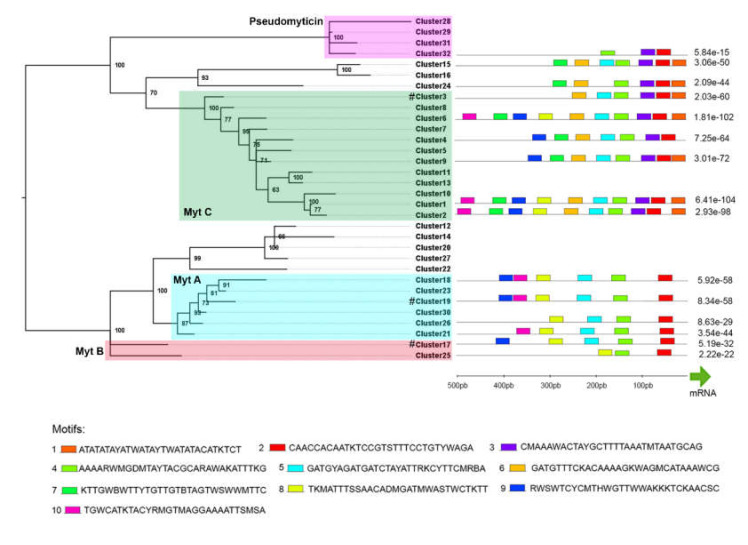
**Promoter analysis.** The 500 bp-long region immediately upstream of the transcription start site, and corresponding to the putative promoter of 15 complete available clusters, was analyzed with MEME. Colored boxes show the 10 different significant motifs found (the sequence of each motif is available in the legend). The p-value derived from the combined observation of all the motifs present in each sequence is also shown. The green arrow marks the beginning of the mRNA sequence. Green (MytC), red (MytB), blue (MytA), and purple (pseudomyticin) squares show the position of the four main described groups of myticins and pseudomyticins. # represents previously published Myt A/B/C sequences.

The promoter analysis allowed us to find 10 different motifs located in the 500 bp upstream of the gene transcription start site, which was expected to include the core and proximal promoter elements (Figure 6). These serial motifs were found in most myticin genes and displayed a nearly invariable position. This observation, together with the high primary sequence divergence among variants, suggests the presence of a well-defined promoter architecture shared by all myticins. Specifically, motifs 2 (CAACCACAATKTCCGTSTTTCCTGTYWAGA) and 4 (AAAARWMGDMTAYTACGCARAWAKATTTKG) were found in all the tested sequences. On the other hand, motifs 1 (ATATATAYATWATAYTWATATACATKTCT) and 3 (CMAAAWACTAYGCTTTTAAATMTAATGCAG) appeared to be associated with each other and were only found in myticin C and evolutionarily-related sequences. Other least conserved motifs were identified in a lower number of sequences, but always displayed high positional conservation.

Although information is currently available about the transcription factor binding sites of any molluscan species in specialized repositories, the strong hemocyte-specificity and high transcriptional activity of myticin genes in physiological conditions [2,12] most certainly suggests that their gene expression is strictly regulated by highly specific and likely unknown transcription factors, which may recognize some of the motifs described above. While we believe our observations may represent genuine transcription factor binding sites candidates, this in silico analysis should be complemented in the future by functional validation, i.e., by the identification of the transcription factors responsible for the regulation of myticin gene expression.

Unfortunately, due to the technical limitations linked with the fragmented nature of the genome assemblies, it was not possible to investigate whether any additional distal regulatory element was associated with myticin genes, either upstream or downstream of the transcription start site.

However, the conserved nature of the myticin promoter prompted us to investigate whether the same motifs could be identified in other genomic regions, associated with the promoter of other AMP gene families (e.g., defensins and mytilins), or with other genes with strong hemocyte-specific expression. However, the screening of the mussel reference genome did not reveal any other gene associated with the 10 aforementioned conserved sequence motifs.

### 3.7. Genomic vs. Transcriptomic data

Having the genome and transcriptome of the same individual offers a great opportunity to investigate RNA editing processes occurring after the transcription [21]. Although eleven different myticin sequence variants were found in the reference genome of LOLA (File S5), only 7 different contigs were present in the transcriptome assembly obtained from the same individual, indicating that four of these were not expressed. The comparison between the genomic and RNA sequences of the seven expressed myticin variants highlighted that there were no discrepancies. This result rules out the possibility that the high level of intraspecific sequence diversity of myticins derives from the mRNA editing process, confirming its genomic origins.

### 3.8. Expression Analysis

**Figure 7:**
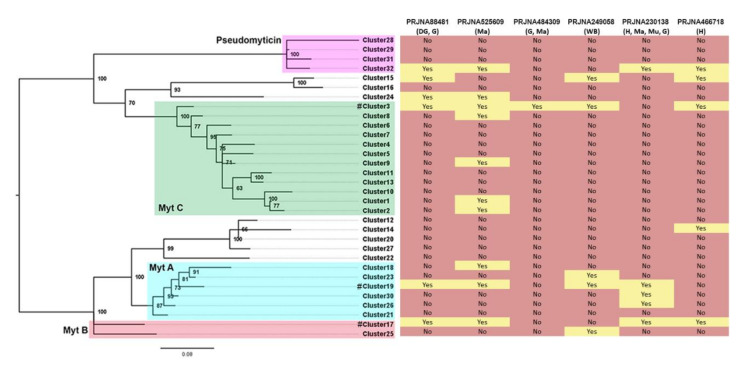
**Expression analysis.** A total of 6 transcriptomes derived from different tissues and mussels sampled in different geographical locations were analyzed to verify the expression of variants belonging to each cluster. The cluster expressed in each transcriptome is highlighted with a yellow background. Red color indicates no evidence of expression. Abbreviations mean: DG, digestive gland; G, gills; Ma, mantle; WB, whole body; H, hemocytes; Mu, muscle. Green (MytC), red (MytB), blue (MytA), and purple (pseudomyticin) squares show the position of the four main described groups of myticins and pseudomyticins. # represents previously published Myt A/B/C sequences.

A total of 6 different transcriptomes deriving from different mussel tissues and geographical locations have been analyzed to investigate whether any of the 32 previously defined sequence clusters were broadly expressed (Figure 7). Again, at least one cluster belonging to the four main groups of myticins (A, B, C, and pseudomyticins) was expressed in all the analyzed transcriptomes (in particular, sequenced from the cluster 3, 17, 19, and 32 were expressed in almost all the analyzed transcriptomes). Some other clusters (most of the cases) were scarcely expressed or even not expressed at all in any of the studied transcriptomes. For instance, clusters 1, 2, 8, 9, 14, 18, 23, and 25 were expressed in only one out of 6 transcriptomes. Some of the clusters lacking evidence of expression are, most likely, pseudogenes, as evidenced by the truncation of the open reading frame due to frameshift or nonsense mutations (Figure 2).

Several of the sequences identified in these transcriptomes showed >5% primary sequence divergence compared with the clusters identified in this study. This observation indicates that the 16 mussels we analyzed, belonging to just two different populations, were not sufficient to build a complete collection of all the possible sequence variants found in the different *M. galloprovincialis* populations across the globe. This suggests that the sequence collection presented in this work might need to be updated in the future with the addition of novel variants and clusters.

## 4. Discussion

Myticins have been traditionally classified in three main groups, myticin A, B, and C. This classification attended to their amino acid sequence and function [1,2]. However, important technological advantages related to massive sequencing techniques have generated additional information that is progressively revealing that the molecular diversity of myticins is much larger than previously thought [21].

The analysis of 16 fully resequenced mussel genomes allowed to ascertain that, on average, each animal possesses 11 different myticin variants, and that a large number of such isoforms are found at very low frequencies in mussel populations. This finding provides strong evidence in support of the hypothesis that each mussel contains its own unique repertoire of myticins, as previously suggested by Costa et al. [3]. These observations are consistent with gene presence/absence variation (PAV), which has been extensively studied in prokaryotes [41] and some plants [42], but only occasionally reported in metazoans. The Mediterranean mussel is the first metazoan where PAV has been described as a widespread phenomenon. Indeed, nearly one-third of the mussel protein-coding genes are subjected to PAV, meaning that they can be either present or absent in different individuals [21]. This study points out that the myticin gene family is also strongly affected by PAV, which appears to be the most important source of intraspecific genetic variation in this case [43]. It is important to note that our phylogenetic analysis revealed that the sequence variability of myticins covered a broad and nearly-continuous spectrum of diversity, preventing precise discrimination of each variant between allelic variants of the same gene and paralogous gene copies. Our clustering approach, based on an arbitrary 95% pairwise identity threshold, therefore needs to be considered with caution, since some of the 32 clusters identified may represent groups of divergent allelic variants. Nevertheless, the high number of variants identified in each mussel, their significant primary sequence divergence, as well as the multi-gene architecture of the myticin gene locus in the reference assembly [21], most certainly indicate that the myticin gene family comprises multiple paralogous genes.

Among the most significant findings of this study, we can report the presence of a new group of peculiar sequences, named pseudomyticins. These encoded peptides are characterized by the loss of the first and fifth cysteines of the typical disulfide array of myticins, which would result in the retention of just three out of the four disulfide bridges described in classical myticins [17]. Despite these unusual features, pseudomyticins appear to be potentially functional genes, as evidenced by the maintenance of conserved motifs in the promoter region, as well as evidenced by their translation to mRNA collected from transcriptome data.

On the other hand, several other myticin variants, which lacked evidence of expression, were characterized by the truncation of the open reading frame, either due to the presence of frameshift/nonsense mutations or due to exon loss. This observation may be consistent with the progressive loss-of-function of some accessory myticin variants generated by past gene duplication events, the fate of which might have headed towards pseudogenization. This phenomenon has been previously observed in mussel mytilins, myticalins, big defensins, and mytimycins [21], as well as in other AMPs from diverse animals whose evolutionary diversification has been driven by gene duplication [44,45].

Like defensins [13,46] and mytilins [40,47], myticins belong to the CSαβ peptide superfamily, which includes several other structurally convergent AMPs found in other domains of life. The conservation of at least three out of the four disulfide bonds in the cysteine array of the mature peptide is essential for the maintenance of the CSαβ structural scaffold. Moreover, most CSαβ peptides have a cationic and amphipathic nature, which is thought to facilitate their electrostatic interaction with the negatively charged surfaces of gram-negative (outer membrane) or gram-positive (cell wall) bacteria [48,49]. The calculated isoelectric point (pI) and net charge at the physiological pH of all myticins were therefore surprisingly low, considering their hypothesized function as AMPs. Compared for instance to mytilins [40], that show an pI between 9–12 and an average net charge of +9, myticins just reached a maximum pI value of 8 and, in most cases, they only had a slightly cationic nature (with a predicted charge ranging from −2 to +4 in cytosolic conditions).

Similar considerations can be extended to the whole sequence of the precursor peptides. Classically, in several AMPs the signal peptide region is neutrally charged, while the mature peptide region displays a strong positive charge, counterbalanced by the negative charge of the C-terminal region [40,49]. However, unlike mytilins and defensins, the pI profile of myticins was quite stable and only showed a significant drop in the charge in the final part of the C-terminal region. These charge distribution properties, unusual for an AMP, would find a justification in the reports that have recently suggested that myticins may cover additional functions, besides pathogen killing. The first indication pointing towards this direction came from the study of Balseiro et al. [5], which proposed myticin C as the first chemokine-like molecule in mussels, but new evidence now supports the chemotactic activity of this molecule. In fact, the expression of myticin was found to significantly increase in mussel after tissue injury, an effect which was not observed in the presence of a pathogen (*Vibrio splendidus*) [19]. Moreover, a correlation between the expression of myticin after a tissue injury and the number of hemocytes recruited at the damaged area was also demonstrated. These observations allowed us to formulate a new functional hypothesis for myticin as a driver of tissue regeneration [20].

The functional importance of cysteines is reflected from an evolutionary point of view. Despite the great variability and complexity of the mussel genome [21] and the enormous intrinsic variability of the myticins, the cysteine array of the mature peptide remained unchanged (with the aforementioned exception of pseudomyticins). Our selection analysis confirmed that this remarkable conservation derives from strong purifying selection. In addition, we provide evidence in support of the strong impact of purifying selection on four additional sites: Ser and Thr residue parts of the alpha helix region, a Gly included in the tight turn connecting the two antiparallel beta sheets and an Arg that limits the C-terminal boundary of mature peptide, which we hypothesize might serve as the site for the proteolytic cleavage of the precursor. In line with a previous report from Padhi and Verghese [50], our analysis revealed the presence of a significant number of sites evolving under diversifying selection. Our approach, with the inclusion of 120 unique sequences and a much higher statistical power, indicates that up to 25% of the sites included in the mature peptide region are subject to positive selection. This observation, supported by the significant overlap with the sites detected with a parallel analysis carried out on myticin C variants only, indicates that positively selected sites are the key sites responsible for the high levels of myticin intraspecific diversity.

Although several studies have previously analyzed the molecular diversity of myticins [2,3,4,16,50], most of these suffered from important limitations, which had so far not been permitted to disclose the basis of these observations. These include: (i) The analysis of cDNA sequences only, which prevented the observation of non-expressed or poorly expressed variants; (ii), the use of PCR amplification, which might have introduced biases with primer design; (iii) the frequent use of data derived from pools of different individuals, which prevented any reliable assignment of variants to individuals; (iv) the lack of paired genomic DNA and mRNA sequence data.

The experimental design of this study avoided all the aforementioned issues and the comparison between the genomic DNA and mRNA sequences obtained from the same individual enabled us to establish that the huge level of sequence variability of myticins has an entirely genomic origin. RNA editing, i.e., the process of post-transcriptional modification of mRNAs through the inclusion of indels or the substitution of nucleotides, common in other mollusks such as cephalopods [51], does not seem to play any role in the generation of sequence diversity in myticins.

In terms of expression, myticins are highly expressed in different developmental stages [52] as well as in mussel hemocytes, where they emerge in the top 10 most actively transcribed genes [2,12]. These observations suggest that a strong core of regulatory elements, including promoters and enhancers, would regulate the expression of myticin genes. How the transcription factors recognize these regulatory elements is far from fully understood [53,54]. In eukaryotic genomes, thousands of genes that encode messenger RNA are transcribed by the RNA polymerase II (POL II) molecular machinery. To initiate the transcription process, RNA polymerase recognizes the promoter region, located immediately upstream of the transcription start site of each gene. Some general motifs recognized by POL II are the B recognition element (BRE), TATA box, initiator (Inr), motif ten element (MTE), and downstream promoter element (DPE) [55]. Although the TATA box is one of the most studied motifs in vertebrates [56,57], other CpG motifs represent other common promoter elements found in vertebrate genomes [58]. In general, TATA boxes tend to be associated with focused transcriptional initiation, whereas CpG motifs tend to display dispersed initiation patterns [59,60]. Even though many eukaryotic core promoters contain some of these motifs, no universal motif has ever been identified as unambiguously present in a core promoter in a given eukaryotic genome [61]. Moreover, to the best of our knowledge, no comprehensive study has ever been carried out to characterize the transcription factor binding sites found in Mollusca or, more broadly, in the Lophotrochozoa superphylum.

With our analysis of the 500-bp sequence upstream of the TSS of myticins, we identified 10 different conserved motifs that may be involved in the regulation of myticin gene expression as core and proximal promoter elements. These motifs were found in a variable number, but in a well conserved order, in canonical myticin genes, as well as in pseudomyticins. Although a putative TATA box could be recognized among these motifs (i.e., motif 1), as described above, no universal promoters have been determined yet. The conserved motifs defined in the myticin promoter are apparently not shared with other mussel genes, including other hemocyte-specific AMPs, like mytilins or defensins, which suggests that the expression of myticins may be controlled by highly specific and still uncharacterized transcription factors. Unfortunately, the limitations posed by the fragmented nature of the individual mussel genome assemblies prevented a detailed characterization of the distal regulatory elements that may contribute to this transcriptional regulation. Taking into account the scarcity of data available about transcription factor binding sites in Lophotrochozoa, the identification of the 10 conserved motifs reported above might provide a solid basis for the identification and functional characterization of the molecular components that determine the high hemocyte-specificity of expression of myticins.

Although previous studies had already indicated that myticins show remarkably high levels of expression, we here provide new evidence that each individual expresses its own repertoire of sequence variants. The combination between evidence of expression and presence/absence at the genomic level demonstrated that just a few canonical myticin clusters were present in the majority of individuals. In contrast, the vast majority of the isoforms are found with low frequency in mussel populations, to the point that we could only identify several of them in a single individual.

## 5. Conclusions

In summary, a total of 120 different myticin variants have been defined and phylogenetically analyzed. All of these variants are subject to presence/absence variation, albeit with different frequency. As expected, the most highly conserved residues of the mature peptide sequence, i.e., the 8 cysteines involved in the formation of the disulfide array, were mostly found to be subjected to strong negative selection, along with a few other previously unreported sites whose functional importance will need to be investigated. While this indicates that deleterious alleles are removed, whenever a non-synonymous mutation occurs in these positions, a high number of other sites (accounting for about 25% of the mature peptide sequence) were found to show signatures of positive selection, which explains the high level of intraspecific sequence diversity observed. The identification of multiple sequence variants in each individual, together with the residual presence of several pseudogenes, further suggests that the molecular diversification of myticins has been made possible by multiple independent gene duplication events.

Most certainly, the data presented in this work indicate that the 120 variants collected from 16 individuals just represent the tip of the iceberg of an underlying extreme level of sequence polymorphism that could potentially reveal several hundred unique myticin variants with follow-up analyses of individuals belonging to populations sampled in other geographical locations.

## Supplementary Materials

The following are available online at https://www.mdpi.com/2218-273X/10/6/943/s1, File S1: Myticin database, File S2: Full genes database, File S3: Reference Genome myticins position, File S4: Phylogeny alignment, File S5: Presence/absence evaluation. The matrix shows the presence and absence of each myticin sequence (120 different sequences) in each mussel genome (16 different genomes), File S6: Isoelectric point analysis. The mature peptide of the whole set of conventional myticins was analyzed. Values of molecular weight, isoelectric point, and charge are available.
